# Structural Studies of the Cutin from Two Apple Varieties: Golden Delicious and Red Delicious (*Malus domestica*)

**DOI:** 10.3390/molecules25245955

**Published:** 2020-12-16

**Authors:** Daniel Arrieta-Baez, María de Jesús Perea Flores, Juan Vicente Méndez-Méndez, Héctor Francisco Mendoza León, Mayra Beatriz Gómez-Patiño

**Affiliations:** Instituto Politécnico Nacional—CNMN, Unidad Profesional Adolfo López Mateos, Col. Zacatenco, Mexico City CP 07738, Mexico; darrieta@ipn.mx (D.A.-B.); mpereaf@ipn.mx (M.d.J.P.F.); jmendezm@ipn.mx (J.V.M.-M.); hfmendoza@ipn.mx (H.F.M.L.)

**Keywords:** cutin, cuticle, *Malus domestica*, CPMAS ^13^C NMR, AFM, CLMS, SEM

## Abstract

The cuticle, a protective cuticular barrier present in almost all primary aerial plant organs, has a composition that varies between plant species. As a part of the apple peel, cuticle and epicuticular waxes have an important role in the skin appearance and quality characteristic in fresh fruits destined for human consumption. The specific composition and structural characteristics of cutin from two apple varieties, “golden delicious” and “red delicious”, were obtained by enzymatic protocols and studied by means of cross polarization magic angle spinning nuclear magnetic resonance (CP-MAS ^13^C NMR), attenuated total reflection infrared spectroscopy (ATR-FTIR), and mass spectrometry, and were morphologically characterized by specialized microscopy techniques (atomic force microscopy (AFM), confocal laser scanning microscopy (CLMS), and scanning electron microscopy (SEM)). According to CP-MAS ^13^C NMR and ATR-FTIR analysis, cutins from both varieties are mainly composed of aliphatics and a small difference is shown between them. This was corroborated from the hydrolyzed cutins analysis by mass spectrometry, where 9,10,18-trihydroxy-octadecanoic acid; 10,20-Dihydroxy-icosanoic acid; 10,16-dihydroxy hexadecenoic acid (10,16-DHPA); 9,10-epoxy-12-octadecenoic acid; and 9,10-epoxy-18-hydroxy-12-octadecenoic acid were the main monomers isolated. The low presence of polysaccharides and phenolics in the cutins obtained could be related to the low elastic behavior of this biocomposite and the presence of cracks in the apple cutin’s surface. These cracks have an average depth of 1.57 µm ± 0.57 in the golden apple, and 1.77 µm ± 0.64 in those found in the red apple. The results obtained in this work may facilitate a better understanding that mechanical properties of the apple fruit skin are mainly related to the specific aliphatic composition of cutin and help to much better investigate the formation of microcracks, an important symptom of russet formation.

## 1. Introduction

Apple production in Mexico was close to 679 thousand metric tons in 2019, up from more than 547 thousand metric tons in 2018 [1]. Usually, the production of fresh apples in Mexico is outpaced by its consumption, and Mexican apple exports are almost residual, with the state of Chihuahua being the main exporter, primarily to the United States (627 metric tons) and Belize (22 metric tons), in the last two years [2].

Apples, like other fruits such as oranges, lemons, grapefruits, strawberries, and others, are covered with continuous, extracellular cuticle [3,4,5]. This cuticle protects the apple against environmental stresses like wind, temperature, chemicals, and drought, not only when attached to the tree, but also after harvesting during storage. Previous research has shown that the cuticle contributes the most to these barrier properties. Without it, apples are prone to infections of mold; physical damage; and, in particular, moisture loss [6,7].

**Scheme 1:**
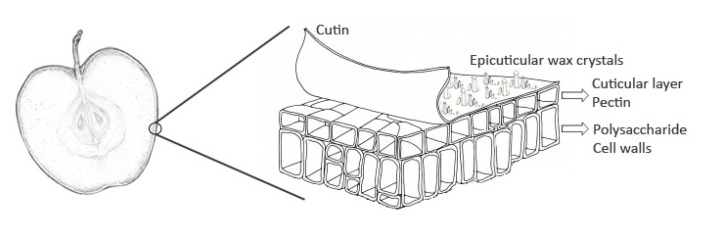
Schematic organization of the fruit cuticle.

Plant cuticles are complex lipid-based biocomposites made of cutin polymer and are filled by non-polymerized cuticular lipids called ‘waxes’ [8] (Scheme 1). Previous reports showed that wax constituents in *Malus domestica* fruits may contain hydrocarbons, alcohols, aldehydes, fatty acids, diols, esters, B-diketones, terpenoids, and phenolic compounds. The importance of these epicuticular waxes has been previously reported.

Cutin is mainly composed of hydroxy- and epoxy hydroxy C16 and C18 fatty acids and glycerol, but its composition can vary among plant species, developmental stages [3,9,10], organs, and environmental stresses [11,12]. This variation in cutin composition affects the structure and properties of the cuticle. For instance, changes in cutin composition have been correlated to plant resistance to the pathogens *Erysiphe polygoni* [13] and *Botrytis cinerea* [14]. Covalent binding of some chemicals can involve specific cutin monomers containing epoxy groups [15] and cutin composition has also been related to the mechanical properties of the cuticle [16]. In fact, hydroxyl groups may enhance the hydrophilic character of the cutin matrix, resulting in a higher elasticity [17]. Lastly, the cutin monomer composition determines the hydroxyl content, and thereby affects cross-linking through polyester bonds [18,19].

Different studies have shown the presence of micro-cracks in the apple cuticle, early in the growing season, and they can be larger with a network formation on the surface at the end of the season [6,20,21]. The presence of cracks makes apple fruits prone to surface disorders such as russeting or skin spots, which results in economic losses [22,23,24]. These disorders have been related to different factors including the wax composition [6,24,25]. However, we believe that a better understanding of the cutin composition would provide more knowledge on the ultrastructure and mechanical properties of the cuticle. In this work, two cutins from two apple cultivars, “golden delicious” and “red”, were obtained and analyzed by means of CPMAS ^13^C NMR, ATR-FTIR, and MS. According to these analyses, both apple cutins are mainly composed of aliphatic components, where the main monomers are 9,10,18-trihydroxy-octadecanoic acid; 10,20-Dihydroxy-icosanoic acid; 10,16-dihydroxy hexadecenoic acid (10,16-DHPA); 9,10-epoxy-12-octadecenoic acid; and 9,10-epoxy-18-hydroxy-12-octadecenoic acid. The low presence of polysaccharides in these cutins could make it susceptible to the formation of micro-cracks observed and analyzed through CLMS, AFM, and SEM.

## 2. Results and Discussion

### 2.1. Cross Polarization Magic Angle Spinning ^13^C Nuclear Magnetic Resonance (CPMAS ^13^C NMR) and Attenuated Total Reflectance Fourier Transform Infrared Spectroscopy (ATR-FTIR) Analysis of “Golden Delicious” and “Red Delicious” Apple Cutins

**Figure 1:**
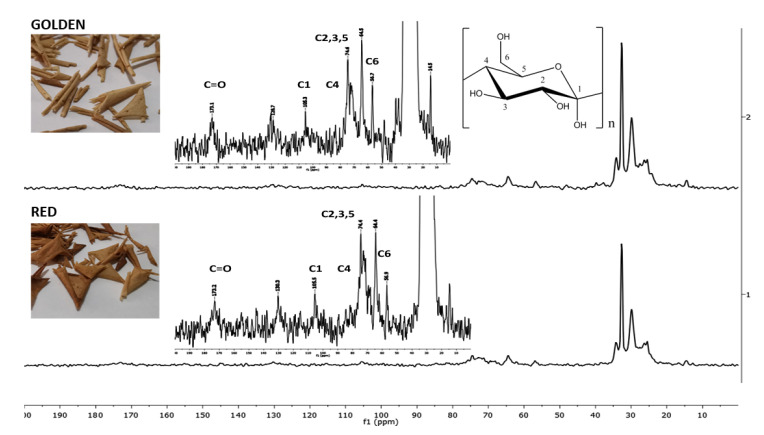
Cross polarization magic angle spinning nuclear magnetic resonance (CPMAS ^13^C NMR) spectra of cutins from “golden delicious” and “red delicious” apples. Besides the carbohydrate peaks assigned, epoxylated compounds were detected because of their signals at δ 56 and 64 ppm.

Apple cutins were isolated according to reported enzymatic treatments [26] and were analyzed by CPMAS ^13^CNMR and ATR-FTIR. For both apple varieties (Figure 1—golden and Figure 1—red), the main resonance assignments were as follows [27]: bulk methylenes (20–40 ppm), which are the most prominent peaks; smaller peaks that are assigned to oxygenated aliphatic carbons (55–85 ppm); aromatics and olefins (105–155 ppm); and carbonyl groups (173 ppm). Besides these groups of signals, those that belong to carbohydrate moieties (C6 at 60 ppm, C2,3,5 at 70–75 ppm, C4 at 83 ppm, and C1 at 105 ppm) can be assigned, even when they are in a very small proportion. Peaks at 56 and 64 ppm are present in both cutins. These peaks are assigned to epoxilated long-chain aliphatic acids (see Supplementary Material S1–S4).

**Figure 2:**
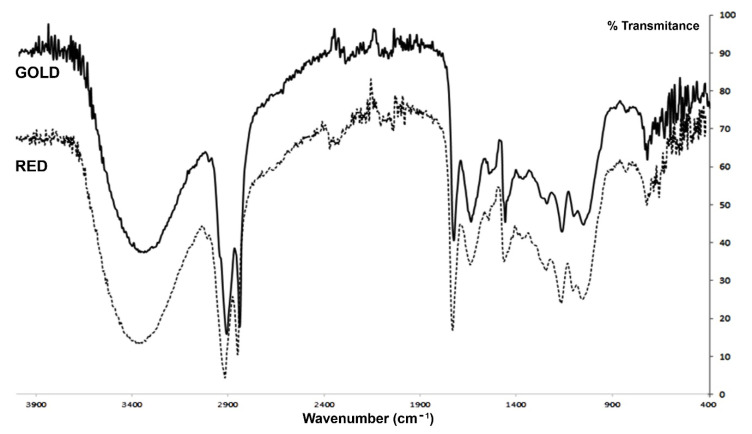
Attenuated total reflection infrared spectroscopy (ATR-FTIR) spectra of cutins from “golden delicious” and “red delicious” apples.

ATR-FTIR spectroscopy has been used to characterize in situ the functional chemical groups of isolated cutins and their interactions with exogenous chemicals at the cuticular level [28,29]. The ATR FT-IR analysis of the “golden delicious” and “red delicious” apple cutins (Figure 2, Golden and Red, respectively) showed a broadband around 3365 cm^−1^ assigned to the stretching vibration of hydroxyl groups of the polysaccharide fraction and residual carboxylic acids, and intense bands at 2918 and 2849 cm^−1^ attributed to the asymmetrical and symmetrical stretching vibrations of CH_2_, accompanied by the corresponding bending vibrations at around 1468, 1313, and 725 cm^−1^.

### 2.2. Direct Injection Electrospray Mass Spectrometry (DIESI-MS) Analysis of the Apple Cutins Alkaline Hydrolysis (KOH/MeOH) Products

To complete the study of both cutins from “golden delicious” and “red delicious” apples, alkaline hydrolysis was practiced by analyzing the main monomers present in this important biopolymer. In both cases, around ≈ 95% of the cuticular material was hydrolyzed. Soluble products from the alkaline hydrolysis were analyzed by means of direct-injection electrospray ionization mass spectrometry in negative mode (DIESI-MS) (see Supplementary Material, S5) and the compounds identified by the *ms*/*ms* analysis are reported in Table 1.

The identified compounds are present in both cutins, with very small differences. The main constituents identified in both cutins were 9,10,18-trihydroxy-octadecanoic acid; 10,20-Dihydroxyicosanoic acid; and 10,16-dihydroxyhexadecanoic acid (10,16-DHPA). 10,16-DHPA, is one of the most important C16 alkanoic acids identified in different cutins such as tomato, citrus cuticles, and green pepper [30]. Two other significant monomers were identified as 9,10-epoxy-12-octadecenoic acid and 9,10-epoxy-18-hydroxy-12-octadecenoic acid in “golden delicious” (10.2 and 4.8%, respectively) and “red delicious” apple cutins (8.26 and 4.18%, respectively). These two epoxylated compounds, along with 9,10,18-Trihydroxy-12-octadecenoic acid and 9,10,18-trihydroxy-octadecanoic acid, could be derived after subsequent oxidation reactions of linoleic acid, detected in the cutin, as well. The predominance of C16 long-chain acids in cutins is common and agrees with previous cutin reports [30]. However, it is important to highlight that most of the 45% of the main monomers in these cutin apples are C18 acids. The presence of these C16 and C18 monomers could be the reason for the broadband esterification detected at 1100 cm^−1^ in the ATR-FTIR spectrum. Aromatic compounds and carbohydrates were not detected by MS analysis in “golden delicious” and “red delicious” apple cutins, maybe because of their low presence and/or their higher polarity, which agrees with the NMR analysis.

### 2.3. Confocal Laser Scanning Microscopy (CLSM) of Apple Cutins

**Figure 3:**
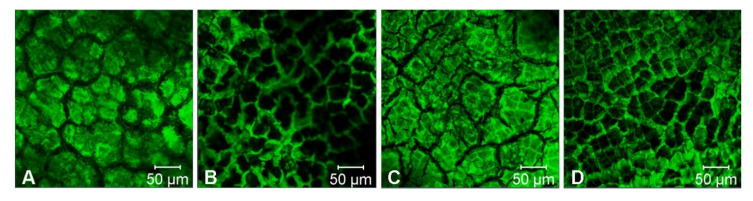
Confocal laser scanning microscopy (CLSM) micrographs of cutins from “golden delicious” ((**A**) outer face and (**B**) inner face) and “red” ((**C**) outer face and (**D**) inner face) apples.

CLSM has been used successfully in the cuticle studies of plant tissues as the cell remains unchanged during the analysis because of the complete absence of pre-treatments or physical sectioning of the samples [31]. In this work, cutins obtained from the two apple cultivars, “golden delicious” and “red delicious”, were analyzed by CLSM. Samples, without artificial staining or physical sectioning, were analyzed directly and most of the structures could be observed, because of their autofluorescence. In fact, the same samples were used in the AFM and SEM analysis to make a better comparison of the structures. The outer surface structure of the apple cutin was visualized as a smooth surface covered with a network of micro-cracks, which agrees with the findings of other research. These micro-cracks seem to be delineated groups of four or more cells, which do not necessarily correspond with the ribs, observed in the inner face (Figure 3).

From Figure 3, the complete analysis of both faces shows that, even when different fractures are observed on the surface, they do not seem to completely penetrate the cutin structure.

### 2.4. Apple Cuticles Characterized by Atomic Force Microscopy (AFM)

**Figure 4:**
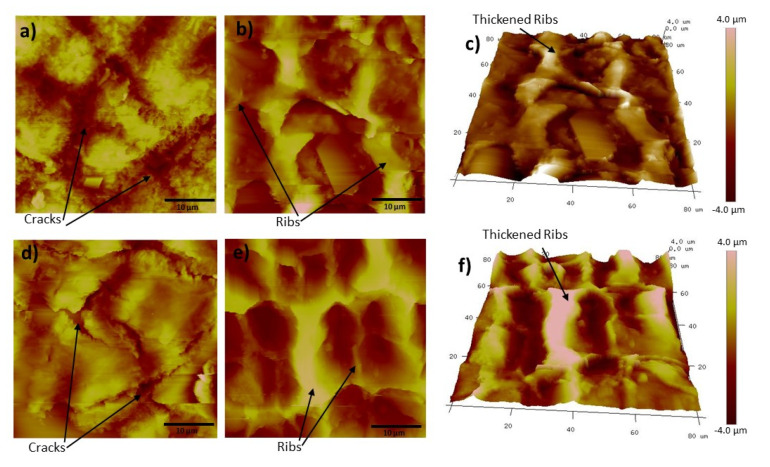
Topographic images obtained by AFM. Figures (**a**–**c**) were obtained from “golden” apple: (**a**) 2D image of the outer face and (**b**,**c**) 2D and 3D images, respectively, from the inner face; Figures (**d**–**f**) were obtained from “red” apple: (**d**) 2D image of the outer face and (**e**,**f**) 2D and 3D images, respectively, of the inner face.

The topography of apple cutins was analyzed by means of AFM (Figure 4). The roughness values for the outer face obtained for “golden delicious” apple cutin were Ra = 533.5 ± 44.5 nm and Rq = 689 ± 45.2 nm, and the values for the inner face were Ra = 1844 ± 69.2 nm and Rq = 2310 ± 79.1 nm, which indicated that the roughness is higher in the inner face; this is because the ribs formed in the cutin conformation in the inner face surface.

In the “red delicious” apple cutin, values of the roughness in the outer face were Ra = 390 ± 44.5 nm and Rq = 486 ± 49.4 nm, and for the inner face, the values of roughness obtained were Ra = 1237 ± 103 nm and Rq = 1514 ± 88.3 nm. Similarly to the “golden delicious” cutin apple measurements, the inner face was the roughest. Moreover, it can be observed from the roughness measurements of both apple cultivars that the “golden delicious” cutin apple is roughest in the outer face, and opposite to the inner face, the “red delicious” cutin apple shows higher values of roughness.

In addition to the roughness differences, topographic differences between the inner and outer faces of both cultivars can be observed (Figure 4). On the external face, structures like islets formed by fissures can be observed, and in the inner face, islets are also observed. However, islets are delimited by thickened ribs similar to walls. The measurements made in the ribs on the inner face of both cutin apple cultivars indicate that the “golden delicious” cutin apple has a height of 3.4 ± 0.7 µm and a width of 10.7 ± 2.9 µm, and in the case of the “red delicious” cutin apple, the height of the ribs is 7.8 ± 2.7 µm and the width is 16.4 ± 5.1 µm. From these measurements, the height and width of the ribs are greater in the “red delicious” cutin apple. These observations agree with those made with CLMS and SEM analysis.

Images obtained by CLMS and AFM showed the particular formation of “islets” or a group of cells delineated in micro-cracks in the outer face of both apple cutins. This cracking is formed during the growth and maturation process of these fruits [7]. During the process, tension stress can be generated in the cuticle, giving rise to the appearance of cracks. In the case of the “red delicious” cutin apple, the cracks are more defined, and their average depth is 1.6 ± 0.6 µm. For “golden delicious” cutin apple, cracks have an average depth of 1.8 ± 0.6 µm and are less defined because they are wider compared with those found in the “red delicious” cutin apple.

### 2.5. Scanning Electron Microscopy (SEM) Analysis of Apple Cutins

The same sample used for CLSM analysis was sputter coated and mounted for SEM analysis. Images were captured at different working conditions, varying the acceleration voltage, working distance, and magnifications. Images were further recorded using secondary electron mode.

**Figure 5:**
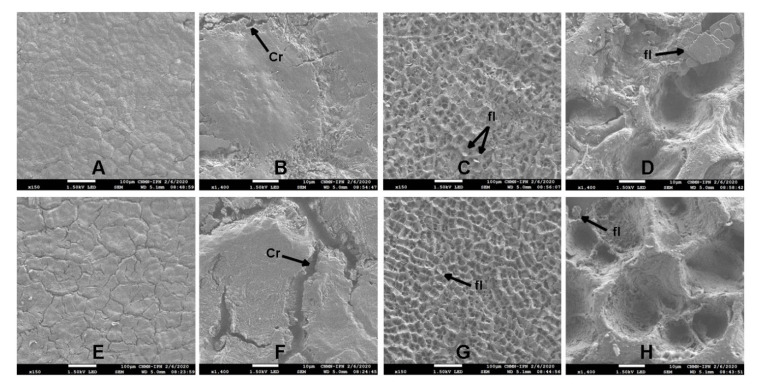
SEM micrographs of cutins from “golden delicious” ((**A**,**B**) outer face and (**C**,**D**) inner face) and “red” ((**E**,**F**) outer face and (**G**,**H**) inner face) apples. Cr: cuticular micro-cracks. fl: epicuticular wax flakes.

Figure 5A,E, show that apple cutin surfaces are covered with an amorphous wax layer characterized by microcracks patterns, which have a considerable variation in length, depth, and distribution pattern, not only between cultivars, but also between sections of the same fruit. According to Figure 5B,F, the zoom of the amorphous wax layer, cracks are deeper and have a greater length in “red delicious” apple cutin. However, they do not extend to the inner face, which agrees with the CLMS analysis. In fact, according to Figure 5C,G, there are no signs of micro-cracks in the inner face. Besides the ribs network in the inner face, epicuticular wax crystals were found in the form of flakes (flat, often polygonal crystals, with distinct edges, that were attached to the surfaces at varying angles, often overlapping like tiles) and platelets (flat crystals without distinct edges, with regular or irregular margins, attached perpendicularly to the surface) (Figure 5C,G). These plates and platelets were irregularly dispersed on the amorphous wax layer inner face and frequently densely concentrated within the microcracks openings (Figure 5D,H). They differed among apple cultivars as well as among cutin apple sections in their width (1.1–5.6 µm), height (0.7–3.2 µm), thickness (0.1–0.4 µm), and density.

The presence of flakes in apple cutins studies using SEM analysis has been reported [7,25]. Even when most authors agree that these flakes are epicuticular wax crystalloids, some of them consider that they are artifacts resulting from the SEM technique, and they cannot be observed with CLMS or another microscopy instrument [31]. According to our previous investigation [32,33], monomers and oligomers derived from tomato and fruit cutins can be in a good organization forming homogeneous films, thus the presence of these flakes could correspond to platelets of aliphatic compounds in the polymerization process.

Besides the biochemical changes throughout the growth process of this fruit, which affect the skin of the apples, mechanical properties of the cutin could be related to the chemical composition affecting the viscoelasticity behavior of the cuticle. Studies with the tomato (*S. lycopersicum*) cuticle have demonstrated that quantitative changes in the cuticle components influence the elastic/viscoelastic behavior of the cuticle [34,35]. Especially, polysaccharides and some phenolics compound like flavonoids, which are related to the rigidity of the cutin network, act as biochemical modulators [35]. The low presence of polysaccharides and phenolics, related to other fruits’ cutins, may affect the elasticity of these apple cutins, initiating a break in the polyesters when the fruit has an expansion during development and ripening.

More studies are needed in order to elucidate the role of polysaccharides in the mechanical behavior of the cutin, and how this can affect its viscoelastic properties.

## 3. Materials and Methods

### 3.1. Chemicals

Trifluoroacetic acid and tissue culture water were purchased from Sigma-Aldrich (St. Louis, MO, USA). The enzymes *Aspergillus niger* pectinase (EC 3.2.1.15), *A. niger* cellulase (EC 3.2.1.4), and *A. niger* hemicellulose (EC 3.2.1.4) were purchased from Sigma-Aldrich (St Louis, MO, USA). Other laboratory chemicals were all of reagent grade or better.

### 3.2. Isolation of “Golden Delicious” and “Red Delicious” Apple Cutins by Enzymatic Methods

Cutin was isolated from the skin of both apple cultivars by a published three-step protocol [26]: (i) peeling and subsequent separation of the cuticle by treatment with pectinase; (ii) enzymatic digestion of cell wall polysaccharides with successive cellulase, pectinase, and hemicellulase treatments; and (iii) exhaustive dewaxing by successive Soxhlet extraction with methanol, methylene chloride, and tetrahydrofuran.

### 3.3. Alkaline Hydrolysis of the Apple Cutins

To 30 mg of cutin, 0.4 mL of deionized H_2_0 and 2.0 mL of 1.5 N methanolic KOH were added; the mixture was fitted with a reflux condenser and stirred at 70–75 °C for 8 or 22 h. Cutin monomers were extracted with CHCI_3_-MeOH using standard procedures [26]. The dried extract was weighed, dissolved in 1.0 mL of CHCI_3_-MeOH (17:3), and filtered through glass wool into autosampler vials. Based on the not hydrolyzed material, the reaction yield was calculated. Typical yields for 8 h alkaline hydrolysis were 90–95%.

### 3.4. Solid-State NMR Spectroscopy Analysis

Insoluble polymers were analyzed using standard CPMAS ^13^C NMR experiments carried out on a Varian Instruments Unityplus 300 wide bore spectrometer (Palo Alto, CA, USA) equipped for solid-state NMR. The resonance frequency was 74.443 MHz, with a customary acquisition time of 30 ms, a delay time of 2 s between successive acquisitions, and a cross-polarization (CP) contact time of 1.5 ms. Typically, each 30 mg sample was packed into a 5 mm rotor and supersonic magical angle spinning (MAS) probe from Doty Scientific (Columbia, SC, USA), and then spun at 6.00 (0.1 kHz and room temperature) for approximately 10 h. No spinning side-bands were observed up- or downfield from the major carbonyl or aliphatic carbon peaks. The resulting data were processed with 50 Hz of exponential line broadening.

### 3.5. ATR-FTIR Spectroscopy Analysis

ATR-FTIR spectra were recorded with an FTIR module IR2 equipped with an indium gallium arsenide (InGaAs) detector (Horiba Jobin Yvon, Longjumeau, France), coupled to a Horiba Jobin Yvon LabRam HR800 spectrometer (Horiba Jobin Yvon, Longjumeau, France). The spectra were recorded in the region of 4000–400 cm^−1^ with a spectral resolution of 4 cm^−1^ and 32 scans per measurement, using an ATR contact objective.

### 3.6. Atomic Force Microscopy Analysis

Images were obtained in the air using a BioScope Catalyst model microscope (Bruker, Santa Barbara, CA, USA) in tapping mode with MESP probes (Bruker, Camarillo, CA, USA). Initially, six cuticles were selected manually, three per face of approximately 8 × 8 mm^2^, and one image 80 × 80 µm^2^ per sample was obtained. Images were analyzed using the Nanoscope Analysis software (Version 1.8, Bruker, Santa Barbara, CA, USA), through which the roughness Ra (arithmetic average of surface roughness) and Rq (the mean square root of surface roughness) were determined. Moreover, measurements of the height and width of the ribs located in the inner faces of both cultivars were made.

### 3.7. Confocal Laser Scanning Microscopy Analysis

For CLSM analysis, each sample was mounted on glass slices and observed under CLSM (LSM 710 NLO, Carl Zeiss, Jena, Germany) with objective EC Plan-Neofluar 10×/0.3. The laser wavelength excitation was 405, 488, 561, and 633 nm, simultaneously. This capture mode used was a spectral imaging technique that automatically outputs separated channels of multiple labeled samples. This tool detects the autofluorescence signal of banana skin and was compared experimentally with patrons (cellulose) between 420 and 720 nm. The z-stack images (3D images) were captured by means of the software ZEN 2010 (Carl Zeiss, Jena, Germany), at 512 × 512 pixels in RGB color, and stored in TFF format at 8 bits.

### 3.8. Direct Injection Electrospray Mass Spectrometry (DIESI-MS) Analysis

DIESI-MS analysis was done on a Bruker MicrOTOF-QII system, using an electrospray ionization (ESI) interface (Bruker Daltonics, Billerica, MA, USA) operated in the negative ion mode. A solution of 10 µL of the sample resuspended in 1 mL of methanol was filtered with a 0.25 µm polytetrafluoroethylene (PTFE) filter and diluted 1:100 with methanol. Diluted samples were directly infused into the ESI source and analyzed in negative mode. Nitrogen was used with a flow rate of 4 L/min (0.4 Bar) as a drying and nebulizer gas, with a gas temperature of 180 °C and a capillary voltage set to −4500 V. The spectrometer was calibrated with an ESI-TOF tuning mix calibrant (Sigma-Aldrich, Toluca, Estado de México, Mexico).

MS/MS analysis was performed using positive and negative electrospray ionization (ESI+/−), and the obtained fragments were analyzed by a Bruker Compass Data Analysis 4.0 (Bruker Daltonics, Technical Note 008, 2004, Billerica, MA, USA). An accuracy threshold of 5 ppm was established to confirm the elemental compositions.

### 3.9. Scanning Electron Microscopy Analysis

Isolated cuticular membranes of apple fruits were mounted on aluminum holders using a conductive double-sided adhesive tape (Plannet Plano), 1 min coated with carbon (60:40) at 25 mA using an SPI sputter coater, and examined in a field emission scanning electron microscope (JEOL JSM-7800F, Tokio, Japan) at 1.5 kV. The sputter conditions, depositing carbon coat thickness of approximately 10 nm, were optimized for the acceleration voltage in the scanning electron microscope.

## 4. Conclusions

Cutins from “golden delicious” and “red” apples were obtained and analyzed. Both apple cutins mainly showed an aliphatic domain according to CPMAS ^13^C NMR and ATR-FTIR. Rendering to these analyses, a very small domain of polysaccharides was detected with no presence of phenolic compounds. The main monomers, characterized as 9,10,18-trihydroxy-octadecanoic acid; 10,20-Dihydroxyicosanoic acid; 10,16-dihydroxyhexadecanoic acid (10,16-DHPA); 9,10-epoxy-12-octadecenoic acid; and 9,10-epoxy-18-hydroxy-12-octadecenoic acid, were identified by DIESI-MS in both apple cutins, with no presence of monosaccharides or phenolic compounds, which agrees with solid-state NMR and ATR-FTIR. The low presence of polysaccharides and phenolic compounds in the cutin could make it susceptible to the formation of micro-cracks during the growth and maturation process. However, more studies are necessary to understand the physicochemical characteristics at this level and correlate it with some physiopathies like russet formation, which result in economic losses.

## Figures and Tables

**Table 1:** Identification of the main compounds in the soluble fraction of the alkaline hydrolysis.

Name	[M-H]^−^_obs_	[M-H]^−^_exact_	Formula	Error	% RA	
					Gold	Red
Palmitic acid	255.2326	255.2330	C_16_H_32_O_2_	−0.8	1.24	2.22
16-hydroxypalmitic acid	271.2268	271.2279	C_16_H_32_O_3_	2.0	6.8	4.92
Linoleic acid	279.2322	279.2333	C_18_H_32_O_2_	−1.3	0.9	1.3
10,16-DHPA	287.2231	287.2228	C_16_H_32_O_4_	−1.1	19.2	15.1
9,10-epoxy-12Z-octadecenoic acid	295.2277	295.2279	C_18_H_32_O_3_	0.4	10.2	8.26
9,10-epoxy-18-hydroxy-12- octadecenoic acid	311.2233	311.2228	C_18_H_32_O_4_	1.1	4.8	4.18
9,10,18-Trihydroxy-12- octadecenoic acid	329.2332	329.2333	C_18_H_34_O_5_	0.5	9.4	8.66
9,10,18-trihydroxy- octadecanoic acid	331.2497	331.2490	C_18_H_36_O_5_	−2.1	20.5	17.68
10,20-Dihydroxy- icosanoic acid	343.2479	343.2490	C_20_H_40_O_4_	−1.1	16.3	11.9

[M-H]^−^_exact_: molecular weight exact, [M-H]^−^_obs_: molecular weight observed, % RA: % relative area. Error [ppm]: absolute value of the deviation between measured mass and theoretical mass of the selected peak in [ppm].

## Supplementary Materials

The following are available online, S1: CPMAS 13C NMR spectrum of the “Golden delicious” apple cutin, S2: Analysis of the “low intensity signals” in the CPMAS 13C NMR spectrum of the “Golden delicious” apple cutin, S3: CPMAS 13C NMR spectrum of the “Red delicious” apple cutin, S4: Analysis of the “low intensity signals” in the CPMAS 13C NMR spectrum of the “Red delicious” apple cutin, S5: Comparative analysis of the DIESI(-) spectra of the hydrolyzed “Golden delicious” (upper spectrum) and “Red delicious” (lower spectrum) apple cutins.
