# Supplementary material for: Structural Studies of the Cutin from Two Apple Varieties: Golden Delicious and Red Delicious (Malus domestica)
Source: Molecules. 2020 Dec 16;25(24):5955. doi: 10.3390/molecules25245955 (PMC7767305; doi:10.3390/molecules25245955)
Supplement: Supplementary file 1 [file molecules-25-05955-s001.pdf]

SUPLEMENTARY MATERIAL

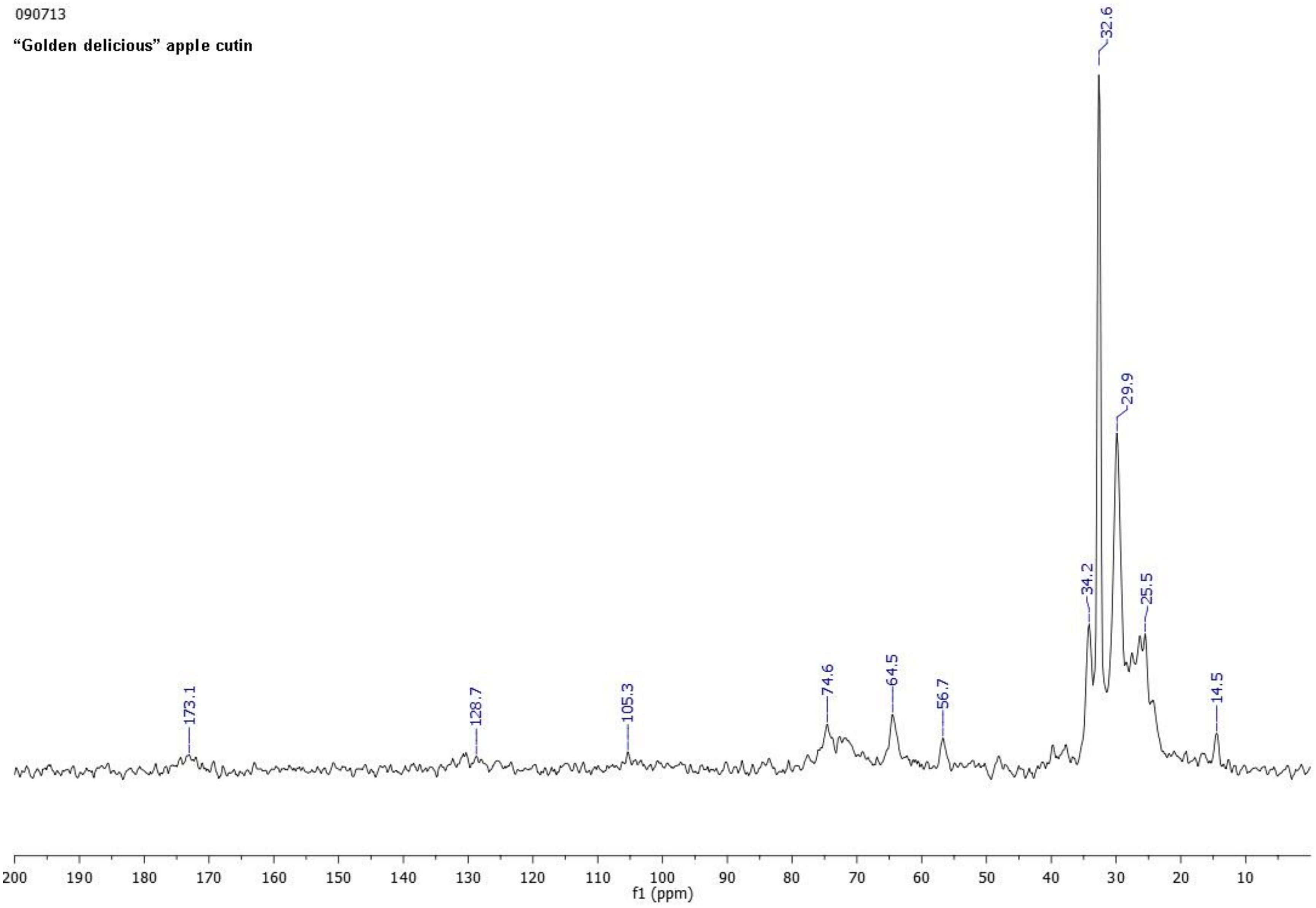

S1: CPMAS 13C NMR spectrum of the "Golden delicious" apple cutin

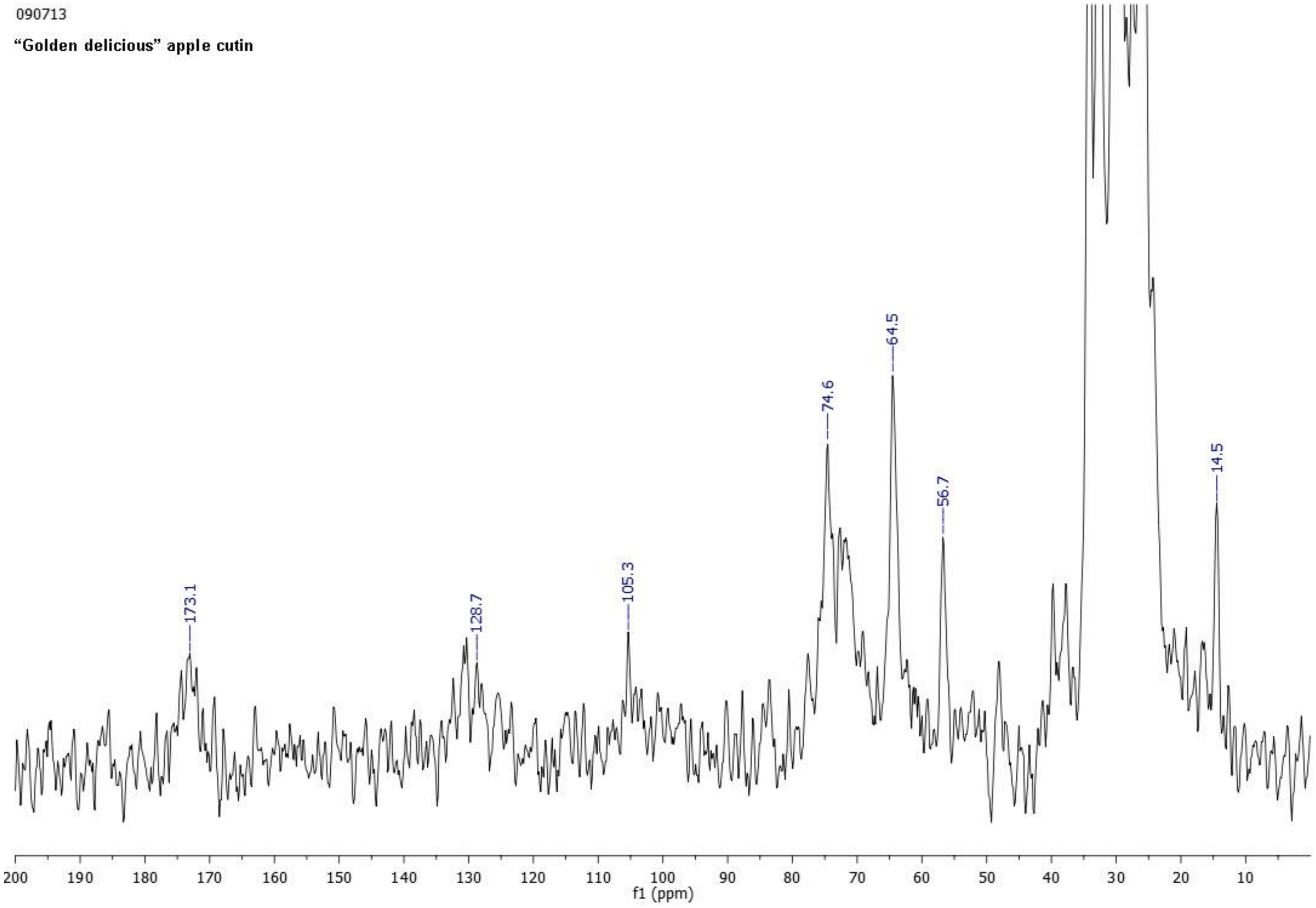

S2: Analysis of the "low intensity signals" in the CPMAS 13C NMR spectrum of the "Golden delicious" apple cutin

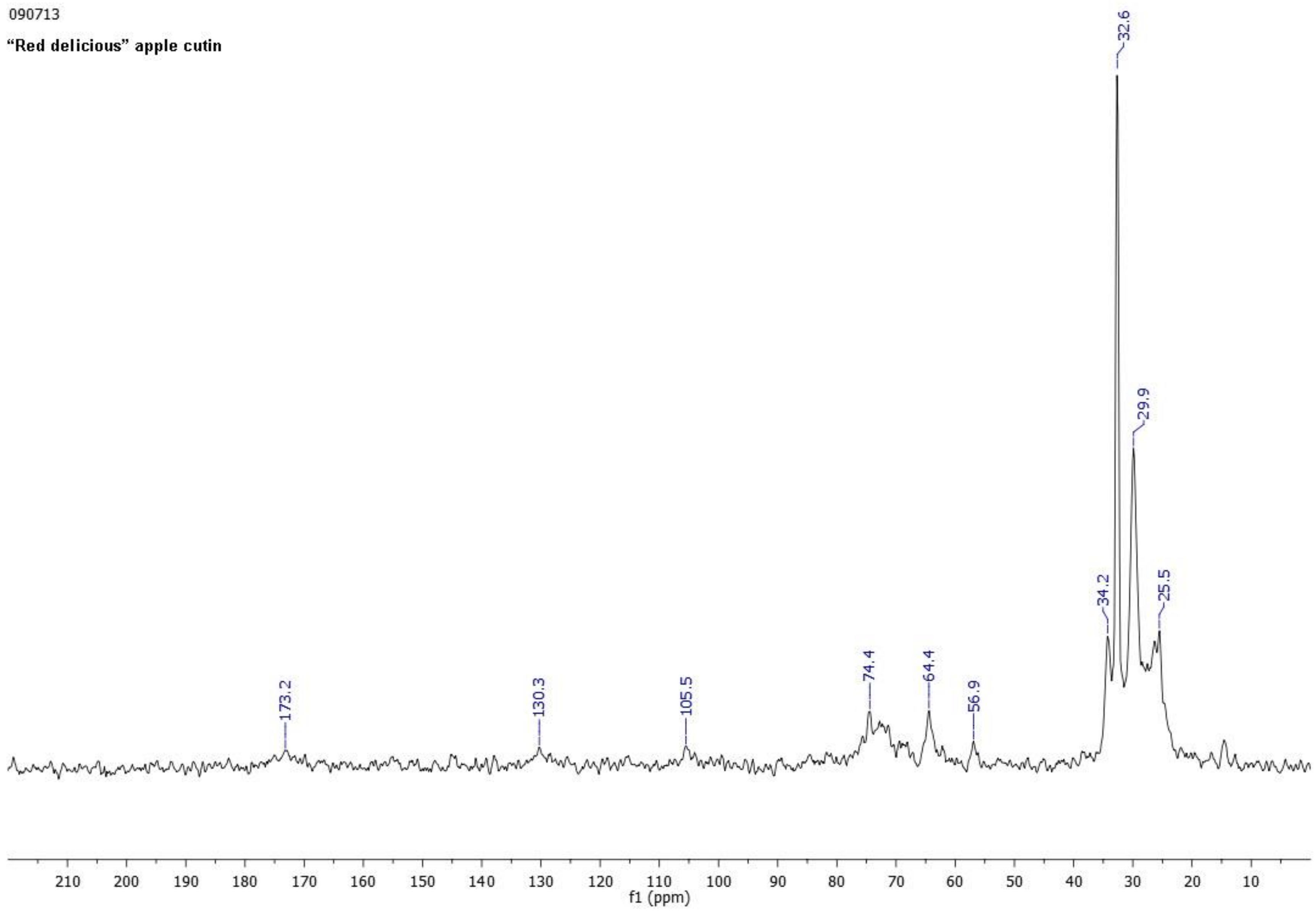

S3: CPMAS 13C NMR spectrum of the “Red delicious” apple cutin

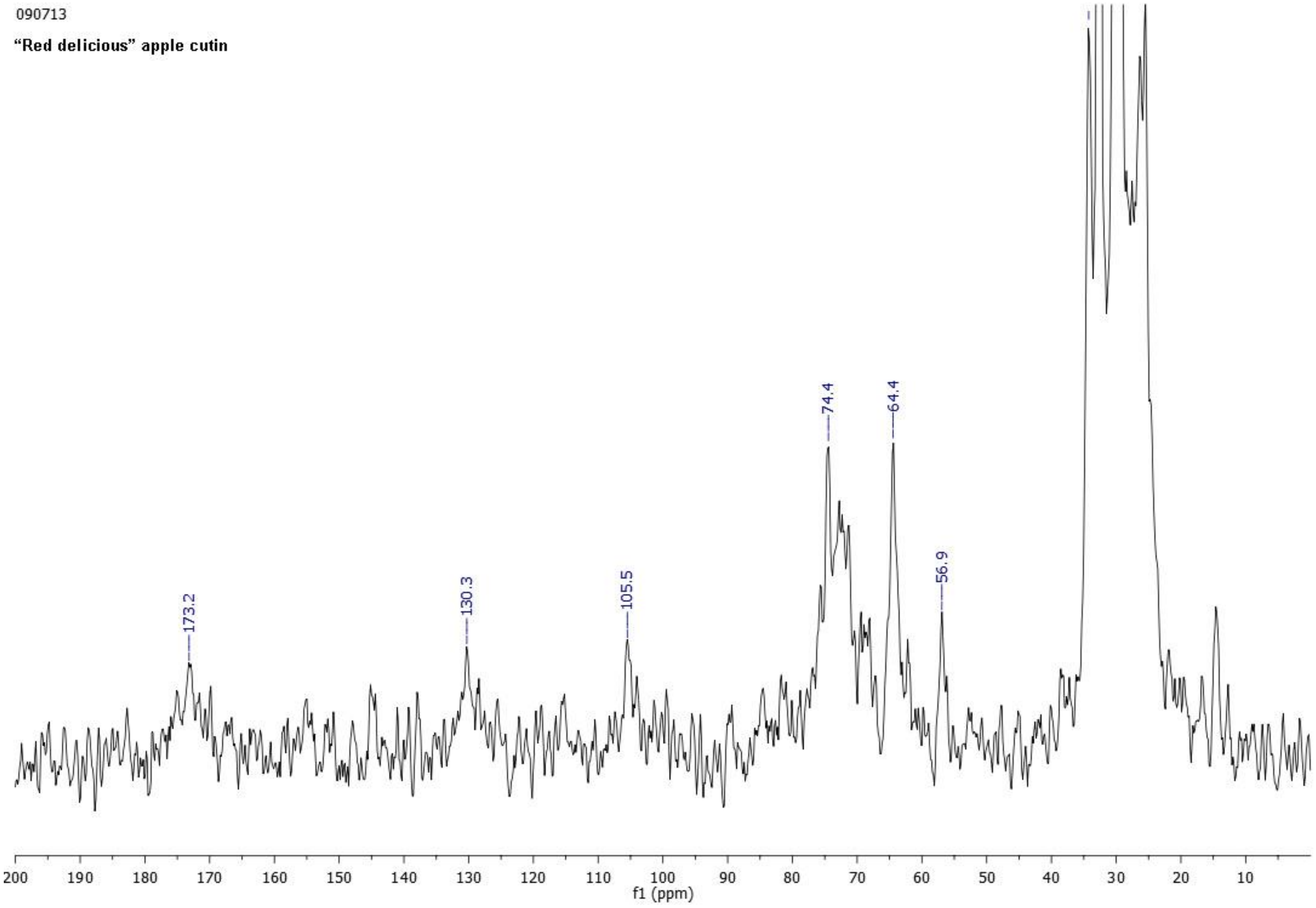

S4: Analysis of the "low intensity signals" in the CPMAS 13C NMR spectrum of the "Red delicious" apple cutin

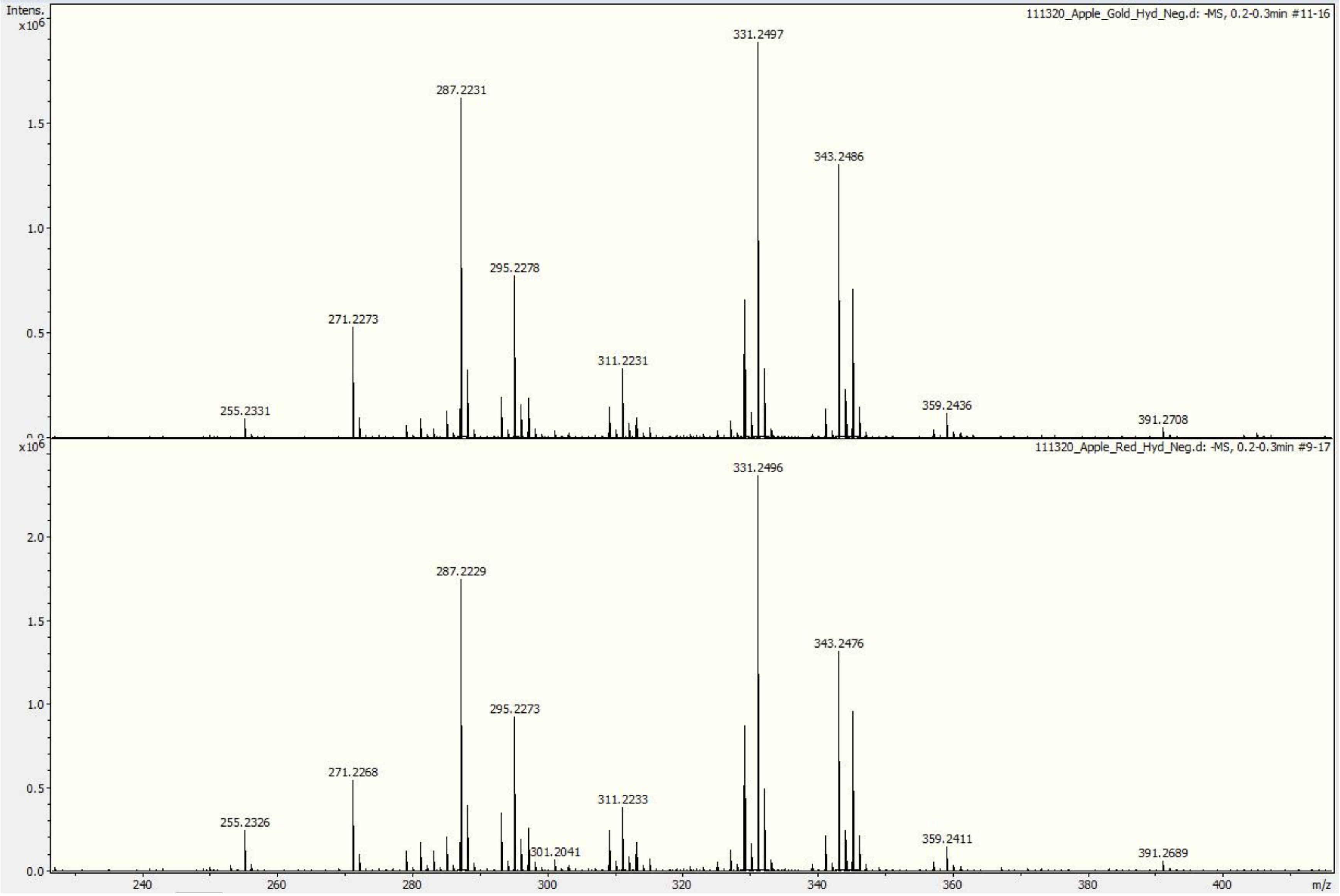

S5: Comparative analysis of the DIESI(-) spectra of the hydrolyzed "Golden delicious" (upper spectrum) and "Red delicious" (lower spectrum) apple cutins.
